# Clinical scale zinc finger nuclease (ZFN)-driven gene-editing of PD-1 in tumor infiltrating lymphocytes (TIL) for the potential treatment of metastatic melanoma

**DOI:** 10.1186/2051-1426-2-S3-P2

**Published:** 2014-11-06

**Authors:** Joal D Beane, Gary K Lee, Zhili Zheng, Nimisha Gandhi, Daniel Abate-Daga, Mini Bharathan, Mary Black, Matthew Mendel, Zhiya Yu, Sadik H Kassim, Smita Chandran, Martin Giedlin, Dale Ando, Ed Rebar, Andreas Reik, Michael Holmes, Philip D Gregory, Nicholas P Restifo, Steven A Rosenberg, Richard A Morgan, Steven A Feldman

**Affiliations:** 1 Surgery Branch, National Cancer Institute, National Institutes of Health, Bethesda, MD, USA; 2 Sangamo Biosciences, Richmond, CA, USA; 3 National Cancer Institute, Bethesda, MD, USA; 4 US National Institutes of Health (NIH), Bethesda, MD, USA; 5 bluebird bio, Cambridge, MA, USA

## 

Multiple inhibitory pathways exist to block the immune response to cancer potentially limiting the effectiveness of adoptive cell transfer (ACT). Programmed cell death-1 (PD-1) is a member of the CD28 superfamily and is expressed on activated T cells. Its ligands, PDL-1 and PDL-2 are expressed on a variety of tumor cells, including melanoma. The binding of PD-1 to PDL-1 inhibits T cell effector function, and represents an important mechanism for PDL-1 expressing tumors to evade the host immune response to cancer. PD-1 thus represents an attractive target for gene-editing of tumor-targeted T cells prior to ACT. To this end, our aim was to eliminate PD-1 expression in tumor infiltrating lymphocytes (TIL) by genome-editing using zinc finger nucleases (ZFNs) directed against the PD-1 gene at a scale sufficient for patient treatment. Using the MaxCyte GT Flow Transfection System to deliver mRNA encoding the PD-1 ZFNs, we show that our clinical scale TIL production process yielded efficient modification of the PD-1 gene locus, with an average modification frequency of 74.8% (n = 3, range 69.9 - 84.1%) of the alleles in a bulk TIL population, which resulted in a 76% reduction in PD-1 surface-expression. Importantly, the PD-1 gene-edited TIL product displayed an effector memory phenotype and expanded approximately 500 - 2000 fold during a rapid cell expansion *in vitro *while retaining T cell effector function. Thus further study to determine the safety of adoptive cell transfer using PD-1 gene-edited TIL for the treatment of metastatic melanoma is warranted.

